# The Calcium-Dependent Switch Helix of L-Plastin Regulates Actin Bundling

**DOI:** 10.1038/srep40662

**Published:** 2017-02-01

**Authors:** Hiroaki Ishida, Katharine V. Jensen, Andrew G. Woodman, M. Eric Hyndman, Hans J. Vogel

**Affiliations:** 1 Biochemistry Research Group, Department of Biological Sciences, University of Calgary, Calgary, Alberta, T2N 1N4, Canada; 2 Prostate Cancer Centre, Southern Alberta Institute of Urology, Rockyview General Hospital, Calgary, Alberta, T2V 1P9, Canada

## Abstract

L-plastin is a calcium-regulated actin-bundling protein that is expressed in cells of hematopoietic origin and in most metastatic cancer cells. These cell types are mobile and require the constant remodeling of their actin cytoskeleton, where L-plastin bundles filamentous actin. The calcium-dependent regulation of the actin-bundling activity of L-plastin is not well understood. We have used NMR spectroscopy to determine the solution structure of the EF-hand calcium-sensor headpiece domain. Unexpectedly, this domain does not bind directly to the four CH-domains of L-plastin. A novel switch helix is present immediately after the calcium-binding region and it binds tightly to the EF-hand motifs in the presence of calcium. We demonstrate that this switch helix plays a major role during actin-bundling. Moreover a peptide that competitively inhibits the association between the EF-hand motifs and the switch helix was shown to deregulate the actin-bundling activity of L-plastin. Overall, these findings may help to develop new drugs that target the L-plastin headpiece and interfere in the metastatic activity of cancer cells.

L-plastin (LPL) is a 70 kDa Ca^2+^- and actin-binding protein that plays an important role in both the adaptive and innate immune system^1^,^2^,^3^,^4^. Among the three isoforms found in humans (L-, T-, and I-plastin), LPL is exclusively expressed in cells of hematopoietic origin, where it can act as an actin cross-linker by promoting actin bundling^5^,^6^,^7^,^8^. The actin-bundling activity of LPL is known to be calcium-dependent, where increasing calcium concentrations inhibit the formation of actin-bundles^9^,^10^. It has been demonstrated that LPL has an optimal Ca^2+^-binding affinity to respond to intracellular Ca^2+^-signals, whereas T-plastin from solid tissues, such as fibroblasts and epithelial cells, is relatively insensitive^11^. The sensitive response of LPL to Ca^2+^-signals is probably crucial for the frequent morphology changes of leukocytes where constant remodeling of the actin cytoskeleton is required to give rise to cell motility. Indeed, LPL is known to be required for T cell activation and motility^2^,^12^. Furthermore, the absence of LPL from neutrophils causes them to be defective and they are no longer able to kill pathogens^13^.

Interestingly, LPL is also expressed after the transformation of a normal cell to a metastatic cancerous cell^14^,^15^,^16^,^17^,^18^,^19^. The protein has also been recognized as a biomarker for the early detection of various forms of cancer, such as kidney^20^, colon^21^, and breast cancer^22^. A calcium signal is induced when cancer cells bind to proteins of the basal lamina^23^. This calcium-mediated signal suggests that LPL plays a crucial role in invasion and cellular adhesion of cancer cells. Indeed, the inhibition of LPL through the intracellular expression of an alpaca antibody blocks filopodia formation and reduces the invasion of prostate carcinoma cells^24^. More recently, studies of the formation of invadopodia and filopodia in cancer cells showed that the two actin-bundling proteins L-plastin and fascin played distinct and complementary roles. However, both proteins were required to create the actin-based cellular protrusions that give rise to the migration, invasion, and the metastatic properties of cancer cells^25^. Furthermore, results obtained with a mouse model have shown that metastasis of prostate cancer is diminished when LPL expression is reduced, while increased LPL expression levels and its phosphorylation give rise to increased metastasis^26^. Therapeutic tools used to decrease the rate of cancer progression have been developed based on recombinant adenoviral vectors that are driven by the LPL promoter^27^,^28^,^29^.

LPL is comprised of two N-terminal EF-hand motifs (head-piece domain), which are homologous to a single domain of calmodulin (CaM) (34.7% identical), followed by two actin-binding domains (ABDs)^5^. Each ABD consists of two independently folded calponin homology (CH) domains. While many signaling and cytoskeletal proteins share these CH domains^30^, plastins are the only known proteins to have two tandem ABDs^31^,^32^,^33^. The four CH domains are each folded into a compact globular shape where the ABD1 and ABD2 are arranged back-to-back^34^,^35^. Each ABD can bind an actin-filament, thereby LPL can cross-link between two actin-filaments to form a tight bundle. Although, it has been shown that ABD1 binds actin ten times stronger than ABD2 *in vitro*^36^, it is still not clear which ABD binds the actin-filament first^36^,^37^.

LPL is regulated not only by the intra-cellular Ca^2+^-concentration, but also by post-translational modifications, such as the phosphorylation of Ser5^22^,^38^,^39^,^40^. Among the three human isoforms, LPL is the only isoform which has been shown to be phosphorylated *in vivo*. Although the protein may have several other putative posttranslational modification sites, to date only phosphorylation of Ser5 (and Ser7 only in hematopoietic cells) has been described in the literature^2^. Phosphorylation at Ser5 of L-plastin allows it to function in a coordinated fashion to permit adhesion, migration, and remodeling of actin to occur^41^. The phosphorylation of L-plastin Ser5 in polymorphonuclear leukocytes is essential for continued adhesion. Jones *et al*. showed that adhesion to serum-coated surfaces was induced by the Ser5-phosphorylated N-terminal peptide of LPS. L-plastin’s targeting to the actin cytoskeleton is mediated by Ser5 phosphorylation^42^. When Ser5, the major phosphorylation site is mutated to Ala, the protein is inactive^41^. However, when Ser5 was substituted with Glu *in vivo*, the mutant protein has the same effect as the native protein^41^. Ser5 phosphorylation is also important for the control of actin turn-over in focal adhesions^43^. On the other hand, the physiological function of Ser7 phosphorylation has not yet been elucidated. Very recently it has been shown that S-glutathionylation may also contribute to the regulation of LPL function^44^.

In addition, LPL activity may also be modulated via direct binding to other proteins such as vimentin^45^, the protein-ionized calcium binding adaptor molecule 1 (Iba1)^46^, grancalcin^47^, or CaM^48^.

Although it has been well established that LPL bundles actin in a calcium-dependent manner, the mechanism of the Ca^2+^-switch is currently unknown. A detailed understanding of the Ca^2+^-switch of LPL at the molecular level could potentially lead to the development of drugs that can deregulate the actin-bundling functions of LPL in cancer cells and prevent them from becoming metastatic. In this study, we have determined the solution NMR structures of the N-terminal headpiece domain, which constitutes the Ca^2+^ sensor EF-hand motif of LPL. The structure was determined in the presence and absence of Ca^2+^. Our original hypothesis was that the headpiece domain would bind directly to one of the four folded CH-domains of LPL, in a manner similar to calmodulin (CaM) binding to CH domains of various proteins^49^,^50^,^51^,^52^. However, this was not the case. Instead, we have discovered that an additional regulatory motif is present in LPL which we have called the ‘switch-helix’. This region is located immediately following the two EF-hand motifs and well before the start of ABD1. It forms an α-helix only in the presence of Ca^2+^, which binds to the EF-hand motifs. Upon removal of Ca^2+^, the switch helix is released and this part of the protein is now unstructured. In subsequent experiments, this region was shown to be important for modulating the actin bundling activity of LPL and that it could act as the Ca^2+^-dependent switch of LPL. In *in vitro* experiments, we have also demonstrated that it is possible to deregulate the Ca^2+^-switch of LPL using a peptide that competes with Helix-5 for binding to the EF-hand domain of the protein.

## Results

### Structural characterization of calcium-free EF and calcium-bound EF-H5

**Figure 1:**
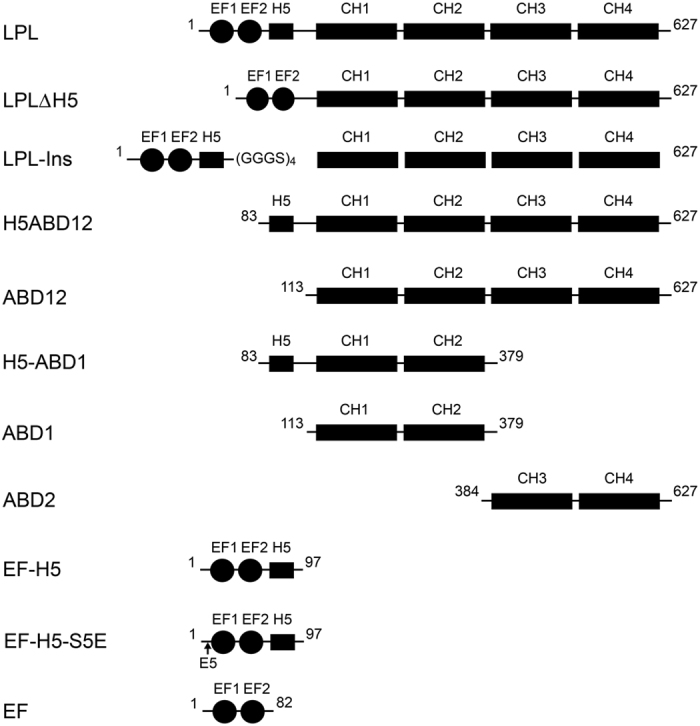
An overview of the various LPL constructs used in this study.

All backbone amide resonances except for the Pro residues of the Ca^2+^-free EF and the Ca^2+^-bound EF-H5 constructs were unambiguously assigned in the ^1^H,^15^N-HSQC NMR spectra (Supplementary Fig. S1). The backbone amide resonances of Ca^2+^-free EF-H5 were also completed except for Arg 91 and Tyr 48, which could not be identified due to extensive signal overlap in the middle of the spectrum. This overlap is characteristic of the presence of some unstructured elements that are present in part of the apo-protein (see below). Next, the chemical shift index (CSI) for the C_α_ and C’ atoms were analyzed to predict the secondary structures in both the Ca^2+^-bound and Ca^2+^-free forms of EF-H5 (Supplementary Fig. S2). As expected, EF-H5 contained four α-helices that make up two helix-loop-helix EF-hand Ca^2+^-binding motifs. Interestingly, the Ca^2+^-bound EF-H5 protein was found to contain an extra α-helix (H5) at the C-terminal end, which was not formed in the Ca^2+^-free form. Taken together with the hetero-nuclear {^1^H}-^15^N NOE data (Supplementary Fig. S2), this region of the protein is unfolded and flexible in the absence of Ca^2+^, while it becomes a stable α-helix upon Ca^2+^-binding. Subsequently, we generated an EF construct in which the H5 region is omitted (Fig. 1; EF construct). We compared the HSQC spectra of the EF and EF-H5 to look for any changes in the structure upon removal of H5 (Supplementary Fig. S3a). As expected, in the Ca^2+^-free form, there are no significant chemical shift changes in the HSQC NMR spectra except for the C-terminal region, indicating that the structure of the apo EF domain is not affected by omitting the H5. Hence, the H5 region is not important for the overall folding of the EF region in the Ca^2+^-free form. By removing this unstructured element from the protein, we could eliminate most of the signal overlap at the center of HSQC spectrum and we could achieve unambiguous assignments that facilitated the determination of the tertiary structure of the Ca^2+^-free form. In contrast, significant spectral changes were observed for the Ca^2+^-bound form of the EF-construct and many signals became somewhat broader (Supplementary Fig. S3b), indicating that H5 is an integral part of the Ca^2+^-bound protein and is a requirement to stabilize its structure.

**Figure 2:**
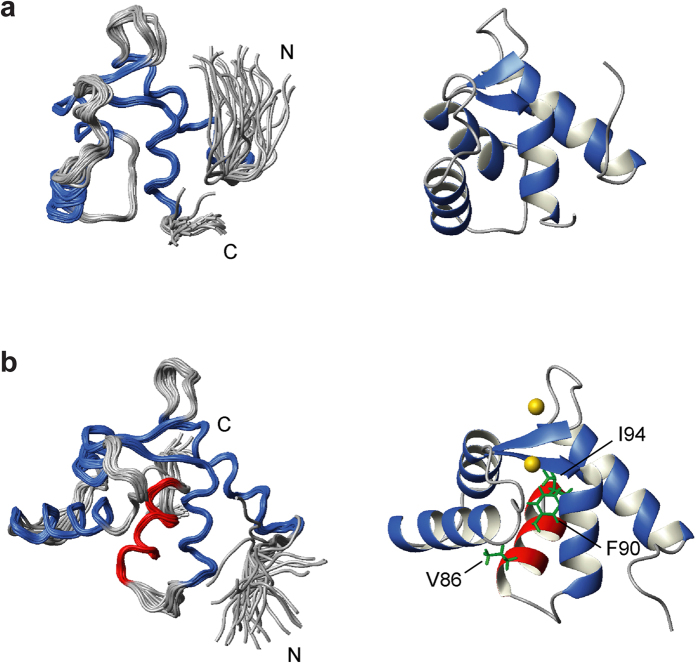
The solution NMR structures of (**a**) Ca^2+^-free EF and (**b**) Ca^2+^-bound EF-H5. The 30 lowest energy structures are superimposed using the backbone atoms in the well folded region (left panels). The four helices which make up the two EF-hands are shown in blue whereas helix 5 (H5) in EF-H5 is shown in red. Ribbon representation of the lowest energy structure (right panels). The key residues (V86, F90, and I94) which associate with the hydrophobic pocket of the EF domain are highlighted. Two Ca^2+^ ions are also displayed as gold spheres.

The tertiary structures of Ca^2+^-EF-H5 and Ca^2+^-free EF were successfully determined using proton-proton distance information derived from NOESY experiments using the automated NOE assignment protocol implemented in the CYANA software. All the parameters from the structure calculations are summarized in Supplementary Table S1. Figure 2a represents 30 NMR solution structures of Ca^2+^-free EF superimposed with a root mean squared deviation (r.m.s.d.) of 0.40 Å. The first EF-hand motif consists of helix 1 (residues 8–21) and helix 2 (residues 31–39). The second EF-hand motif consists of helix 3 (residues 48–60) and helix 4 (residues 71–79). The two calcium-binding loops of the EF-hand motifs are connected through a small anti-parallel β-sheet, as is typically found in such proteins^53^,^54^. The bundle of four helices is in a closed conformation where the hydrophobic pocket is hidden inside; this arrangement closely resembles the isolated N- and C-domains of Ca^2+^-free CaM. In Fig. 2b, the superimposed 30 structures of Ca^2+^-bound EF-H5 are displayed with a rmsd of 0.37 Å. Similar to the Ca^2+^-free form, the EF-hand motifs are composed of helix 1 (residues 8–21), helix 2 (residues 31–40), helix 3 (residues 47–57), and helix 4 (residues 71–79), except that the bundle is now in an open-conformation. Ca^2+^-binding induces dramatic changes in the angles between the four helices, resulting in the exposure of a hydrophobic pocket (Supplementary Fig. S4). Interestingly, an extra helix (H5; residues 85–94) is tightly associated with this exposed pocket, which is very similar to a typical CaM-target peptide interaction^55^. Bulky hydrophobic residues of H5 including V86, F90, and I94 are key residues that associate with the hydrophobic pocket and act as anchoring residues (Fig. 2b). The EF-hand domain and H5 are directly connected by a short flexible linker (Fig. 2b and Supplementary Figs S2). As the H5 region is free from the EF domain in the absence of Ca^2+^, we wondered if this region becomes a part of the ABDs. We have compared the ^1^H,^15^N HSQC NMR spectra of ^15^N-labeled ABD1 (residues 113–379) and H5-ABD1 (residues 83–379) (Supplementary Fig. S5a). There are no substantial chemical shift differences except for extra signals in the H5-ABD1 spectrum all of which are located in a random coil region of spectrum (~8 ppm). Next, we investigated the possible interaction between H5 and ABD2. We titrated the synthetic H5 peptide into the sample containing ^15^N-labeled ABD2 (residues 384–627) (Supplementary Fig. S5b). Again, no chemical shift changes were observed, indicating no interaction. Therefore, we have concluded that in the absence of any actin filaments, the H5 region is free from either the EF-domain or the ABDs in the absence of Ca^2+^.

### Effect of Ser5 phosphorylation on the EF structure

In order to study the structural effects caused by the Ser5 phosphorylation, we have prepared a S5E mutant of the EF-H5 construct. The S5E mutation has been utilized previously to mimic the phosphorylation at Ser 5^41^. Our results showed that when Ser5 was mutated to Glu, both the Ca^2+^-free and Ca^2+^-bound form experienced only a slight change in conformation, which can be seen from the NMR spectra (Supplementary Fig. S6). The few residues that experience chemical shift changes were all located in the immediate vicinity of the mutation. Therefore, phosphorylation does not seem to affect the interaction between the EF domain and H5 and the global structure of the EF domain remains unchanged. This result is resonable as this phosphorylation site is located in the flexible N-terminal portion of EF-H5 in both forms (Supplementary Fig. S2).

### Isothermal titration calorimetry (ITC) experiments

**Figure 3:**
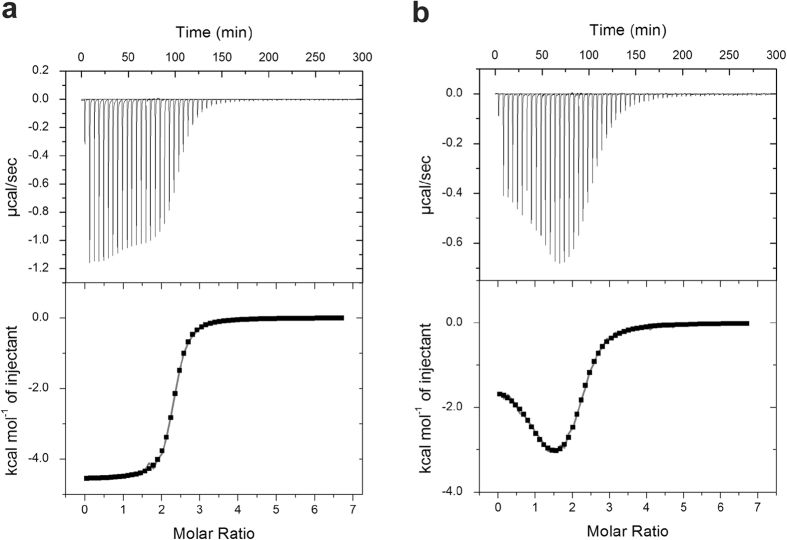
Calorimetric titration of (**a**) EF-H5 and (**b**) EF with Ca^2+^. The baseline corrected ITC titrations are shown in the top panel. Derived binding isotherms were fitted to obtain thermodynamic parameters (bottom panel).

To investigate the role of H5 in Ca^2+^-binding to the Ca^2+^-sensor domain of LPL, we performed ITC experiments for the EF and EF-H5 constructs. Figure 3a shows the ITC isotherm obtained during a calcium-titration experiment and the fitting to determine the thermodynamic parameters for EF-H5. The isotherm shows that it contains two binding events which are very close in their Kds. However, we were not able to unambiguously derive two sets of ITC parameters from these data. The first Ca^2+^ binding seems to produce slightly larger heat, which may possibly be attributed to the folding of H5. When we analyzed these data as a single binding event, a Kd value of 0.7 ± 0.2 µM (range indicates SD, *n* = 3) with a stoichiometry of approximately 2 was obtained and this is consistent with a previous report^11^. On the other hand, the EF construct in which the H5 region is omitted showed two clearly distinctive sequential Ca^2+^-binding events (Fig. 3b). In this case, the curve fitting with a two sites model produced two very different Kd values, 0.3 ± 0.1and 2.6 ± 0.2 µM (ranges indicate SD, *n* = 3) that could easily be fitted. These results suggest that H5 is required for promoting high affinity Ca^2+^-binding to one of the two Ca^2+^-binding sites. These data are summarized in Table 1.

### Actin-bundling assay with various LPS constructs

**Figure 4:**
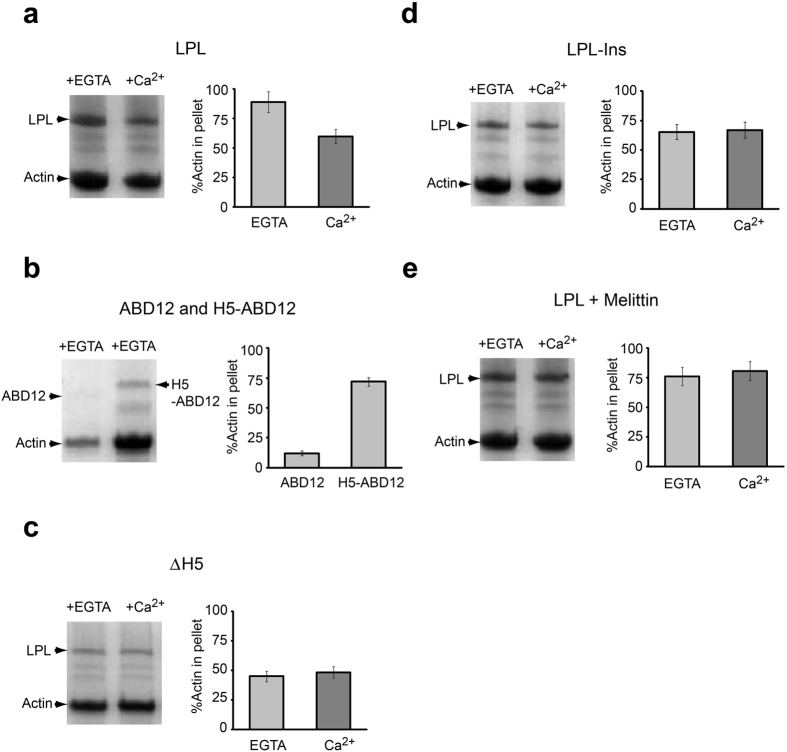
Actin-bundling assay with various LPL constructs in the presence of Ca^2+^ or EGTA. After low speed centrifugation, the amount of actin in the pellet was estimated via SDS-PAGE and Coomassie brilliant blue staining. Each experiment was repeated three times (*n* = 3) to obtain average values and standard deviations (SD).

In order to investigate the role of H5 in actin-bundling, we incubated platelet actin and various LPL constructs (depicted in Fig. 1) to form actin-bundles and we analyzed the amount of actin in the pellet after low-speed centrifugation (Fig. 4). The full-length LPL shows Ca^2+^-dependent actin-bundling where ~90% of the actin was found in the pellet in the presence of EGTA, whereas the amount was reduced to ~50% in the presence of Ca^2+^ (Fig. 4a). Consistently, ~70% of LPL was found in the pellet with EGTA, while this amount was reduced to ~50% with Ca^2+^ (Fig. 4a). This result is in line with previous reports^9^. It was unexpected that the ABD12 construct, which only contains four CH-domains, produced only a very small amount of actin bundles (Fig. 4b). However, the inclusion of H5 in the construct (H5-ABD12) dramatically increased the amount of actin bundles to almost the same level with the full-length LPL, suggesting that H5 is indispensable for actin-bundling of LPL. Consistently, with the LPL constructs in which the H5 region was deleted (LPL-ΔH5), the amount of actin bundles was drastically reduced particularly in the presence of EGTA (Fig. 4c). Next, we have tested the LPL-Ins construct where an extra 16 amino-acid residues were inserted to separate H5 from the ABDs (Fig. 1). Similar to LPL-ΔH5, less effective actin-bundling was observed in the presence of EGTA (Fig. 4d).

### Surface plasmon resonance (SPR) experiment

SPR experiments were carried out to investigate the interactions between the EF domain of LPL (EF construct) and various synthetic CaM-binding peptides. Since the EF structure of LPL is very similar to one of the two CaM domains, we decided to study two typical CaM-binding peptides, CaMKIp and smMLCKp, as well as the cytotoxic peptide melittin, which is also known to bind to CaM^56^,^57^ (Supplementary Fig. S7). Although, all peptides tested were able to interact with the EF domain of LPL in the presence of Ca^2+^, the quality of the SPR data obtained for the smMLCKp was not sufficient for the analysis (data not shown). The Kd values derived were compared to that of an H5 synthetic peptide representing the H5 region of LPL (residues 83–100). The Kd’s obtained are listed in Table 2. The affinity between EF and the H5 peptide was found to be relatively weak with a Kd value of 3.5 × 10^−6^ M. This is consistent with the intermediate exchange and resultant line broadening observed in the HSQC NMR spectrum when the H5 peptide is added into ^15^N-labeled EF (Supplementary Fig. S8). The strong CaM binding peptides (Kd = <10^−8^ M), CaMKIp and smMLCKp, could also bind to the EF domain of LPL, albeit weakly. Interestingly, the melittin peptide bound one order of magnitude stronger than the H5 peptide.

### Interactions between LPL and melittin, and its effect on actin-bundling

**Figure 5:**
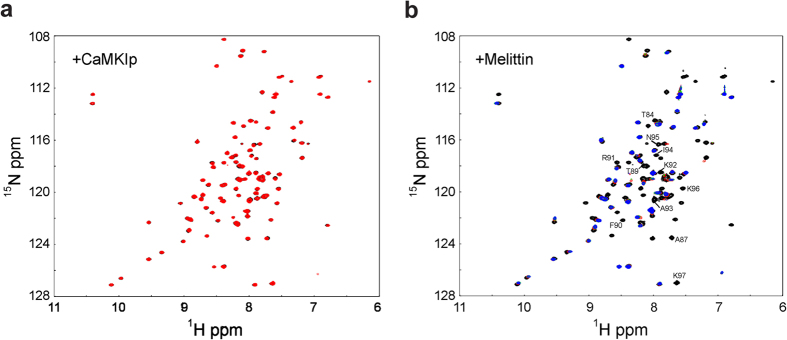
Ca^2+^-EF-H5 is titrated with the CaMKIp or melittin peptide. ^1^H,^15^N HSQC NMR spectrum of EF-H5 (black) is overlaid with the spectrum of EF-H5 with 2 molar excess of CaMKIp (red) (**a**) and with 0.5 (red), 1.0 (green), and 1.5 (blue) molar excess of melittin (**b**). The assignments for the affected signals are indicated.

Since we have found that melittin can bind stronger to EF than the synthetic H5 peptide, we were interested to determine if this peptide can displace H5 from the Ca^2+^-bound LPL. We titrated the melittin peptide into a sample containing the ^15^N-labeled EF-H5 and the chemical shift changes were monitored by HSQC NMR spectra (Fig. 5). Many signals disappeared as we added the peptide, indicating an interaction with an intermediate exchange behavior on the NMR time scale. As we expected, most of the signals that disappeared originated from the residues in the bound H5 region (Fig. 5), suggesting that H5 is displaced by the melittin peptide. As a control, a titration experiment was also performed with the CaMKIp peptide, which has almost the same Kd as the H5 peptide (Table 2). There were no substantial chemical shift changes detected in this titration, indicating that the CaMKIp peptide was unable to displace H5. Finally, we have examined whether the presence of melittin peptide affects the actin-bundling of LPL (Fig. 4e). The Ca^2+^-dependent regulation of actin-bundling of LPL was clearly less pronounced in the presence of melittin. The actin-bundling induced by the melittin peptide alone was negligible (data not shown).

## Discussion

By determining the NMR solution structures of the EF-hand domains of LPL, we have discovered that an extra ‘switch-helix’ can be formed between the EF construct and the ABDs which may act as the Ca^2+^-sensor. This fifth helix of LPL (H5) is only formed in the presence of Ca^2+^ when it is bound to the two EF-hand motifs. On the other hand, the H5 region is released from the EF domain and becomes unstructured in the absence of Ca^2+^. This is consistent with the previous CD spectroscopy results that show a large increase in the helical content in EF-H5 upon binding Ca^2+^ ions^11^. H5 is tightly associated with the exposed hydrophobic pocket of Ca^2+^-bound EF domain, which is reminiscent of CaM-target peptide binding^55^. Interestingly, in contrast to the methionine rich hydrophobic pockets of CaM, all four methionine residues of EF-H5 are located on the outside surface of the LPL EF domain (Supplementary Fig. S4). The flexible side chains of methionine allow the target binding pockets of CaM to accommodate widely different targets^55^. This implies that the hydrophobic pocket of the LPL EF-hands is devoid of methionine side-chains and seems to be designed to be rather specific for the H5 sequence. Importantly, amino-acid sequence alignments show that the H5 region is highly conserved among all three human plastin isoforms (data not shown). Furthermore, from our sequence analysis, the plastin isoform from the amoeba *Dictyostelium discoideum* (FimA) also appears to have a potential H5 helical region and it has been shown that this protein can also bundle actin filaments in a Ca^2+^-dependent manner^58^. LPL represents an example of an automodulation mechanism that is seen in some EF-hand proteins such as plant Ca^2+^-dependent protein kinases (CDPKs) and the ER Ca^2+^-sensor stromal interaction molecule 1 (STIM1), where the calcium sensor domain and regulatory target domain are integrated in the same protein^54^.

The H5 region of LPL was previously predicted as a potential binding site for CaM^48^. Indeed this sequence produced a high theoretically predicted score for CaM binding^59^. Wabnitz and coworkers have demonstrated that CaM and LPL co-localize in the T-cell/APC contact zone and that the deletion of the H5 region decreases the localization level of LPL by ~50%. In their work, the direct binding between CaM and LPL was confirmed by a CaM-pull down assay, and it was only seen in the absence of Ca^2+^. Therefore, we have tested the interaction between EF-H5 and CaM in the presence of EDTA. However, we were unable to detect any substantial chemical shift changes in the HSQC spectrum of ^15^N-labeled apo-CaM when we added non-labeled EF-H5 (Supplementary Fig. S9). These data suggests that the interface for CaM should be located outside of the EF-H5 region (possibly in the CH-domains). Indeed, CaM binding to CH-domains from many different actin-binding proteins has been reported before^49^,^50^,^51^,^52^. The decreased localization level of LPL in the contact zone by removing the H5 region may then be attributed to the reduced actin-bundling activity of LPL (see below).

**Figure 6:**
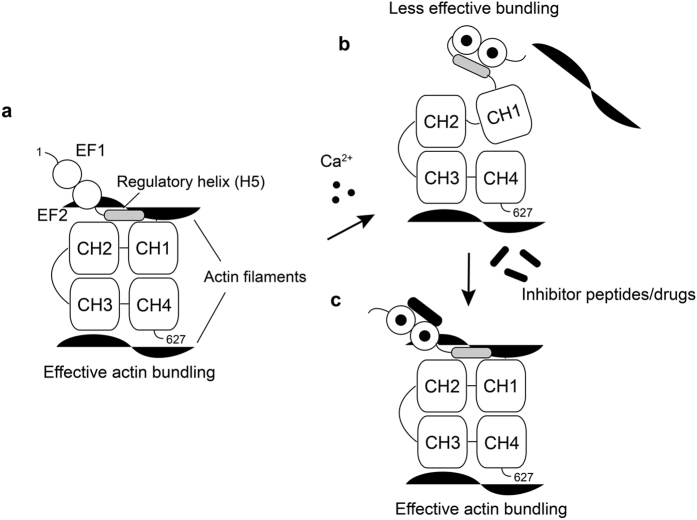
Possible model for the regulatory Ca^2+^-switch of LPL. (**a**) The regulatory helix (H5) stabilizes the actin binding interface formed with CH1 and 2 (ABD1) in the absence of Ca^2+^. (b) The Ca^2+^-bound EF hands sequesters H5 from ABD1, resulting in a less stable domain orientation, which leads to the lower actin bundling efficiency. (c) Peptides or drugs which can block the association between H5 and EF hands can potentially deregulate the Ca^2+^-switch of LPL.

In order to further investigate the role of H5 in the Ca^2+^-dependent actin-bundling of LPL, we have performed actin-bundling assays with a series of different LPL constructs (Fig. 4). The deletion of H5 from LPL (LPLΔH5) significantly reduced the bundling activity of LPL in the presence of EGTA compared to the wild-type, suggesting that H5 plays an important role in this activity (Fig. 4a,c). However, the activity also dropped slightly in the presence of Ca^2+^ compared to the full-length LPL, suggesting that possible steric hindrance could be caused by the reduced distance between the EF domain and the ABDs. Therefore, in this work, we also generated the LPL-Ins construct (Fig. 1). By replacing the H5 in LPL with a (GGGS)_4_ linker, the actin-bundling activity also dropped substantially in the absence of Ca^2+^, albeit to a lesser extent than that seen with the LPLΔH5 (Fig. 4d). This observation confirmed that the steric hindrance caused by the reduced distance is not the major reason for the reduced actin bundling. We noted that our results indicated that the bundling activity of the isolated ABDs (ABD12) was very poor (Fig. 4b), however, the slightly longer construct that also contained H5 (H5-ABD12) dramatically increased the amount of bundled actin. From these results, it seems reasonable to conclude that H5 is required for sustaining the effective actin-bundling activities of LPL and that the Ca^2+^-bound EF domain sequesters H5, which therefore acts as a Ca^2+^-switch. Previous cryo-EM studies of LPL constructs, when they are decorated on the F-actin filament, have provided significant low resolution structural information^37^,^60^,^61^,^62^,^63^. Although isolated ABD1, which consists of CH1 and CH2, binds an order of magnitude stronger than ABD2, containing CH3 and CH4^36^, cryo-EM studies have shown that ABD1 binds to actin filaments in a polymorphic and sparse manner^37^. Indeed, the crystal structure of the actin-binding core (ABDs) of *Arabidopsis thaliana* fimbrin (AtFim1) and *Schizosaccharomyces pombe* fimbrin (sac6) have revealed that the relative domain orientation between the CH1 and CH2 cannot be defined with a single orientation, which suggests some flexibility between the domains^34^. Indeed, the relative domain orientation in the crystal structure was not consistent with that determined by the cyro-EM studies^60^,^61^,^63^ and was not supported by previous mutational studies^64^. These observations suggest that a significant rearrangement of relative domain orientation of the four CH domains is required prior to actin-binding. Based on the earlier cryo-EM results and our own observations, we propose a possible model for how the actin-bundling of LPL is regulated by Ca^2+^ (Fig. 6). When the intra-cellular Ca^2+^-concentration is low, the H5 region is free from the EF domain. According to our results, H5 does not interact directly with the ABDs in the absence of any the actin filaments (Supplementary Fig. S5). However, from the cryo-EM structure^60^, the H5 region may interact with ABD1 (between CH1 and CH2) upon binding to the actin-filament where H5 acts as a wedge to stabilize domain orientation to form a favorable actin-binding interface. When the Ca^2+^-concentration is elevated, the Ca^2+^-bound EF-domain sequesters H5 from the ABD1 and the removal of the wedge would lead to an unstable domain orientation and therefore lower the actin-bundling efficiency. The possible sterical hindrance caused by the reduced distance between EF and ABDs may play a role in the regulation as discussed above. It has been shown that ABDs become more resistant to tryptic digestion in the presence of Ca^2+^, suggesting that the ABDs are protected by the spatially close EF domain^60^. In this study, we have also found that H5 is not only the Ca^2+^-switch of actin-bundling, but also an important modulator for the Ca^2+^-binding to the EF domain. The association of H5 with the EF-hand motifs is required to enhance the Ca^2+^-affinity of one of Ca^2+^-binding sites to µM range. Very recently, a similar role for a helix that is positioned in the vicinity of the EF-hand motifs has been reported for one of the NCS family calcium binding proteins, the guanylyl cyclase activator protein 1 (GCAP1), in which an adjacent helix is also important for high affinity Ca^2+^-binding^65^.

Since we have established a model for the Ca^2+^-switch, it was of great interest to us to determine whether peptides/drugs can out-compete H5 and consequently disable the Ca^2+^-switch of LPL (Fig. 6c). This may lead to the development of potential drugs that could contribute to suppressing the metastatic activity of cancer cells. We have performed SPR experiments with various CaM-binding peptide candidates (Supplementary Fig. S7). The synthetic peptide corresponding to the H5 region binds to the Ca^2+^-bound EF with the Kd of 10^−6^ M. Considering that H5 is directly linked to the EF domain in the actual protein, it would require much higher affinity to outcompete. Indeed, the HSQC spectrum of ^15^N-labeled EF saturated with the H5 peptide did not produce the same spectrum as EF-H5, suggesting a much lower affinity than when they are directly connected (Supplementary Fig. S8). Among the peptides we have examined, only melittin showed a much higher affinity than the H5 peptide (Table 2). We have confirmed that melittin can out-compete H5 in the EF-H5 construct (Fig. 5). Melittin also reduced the Ca^2+^-dependency of the actin-bundling of LPL, indicating that this peptide can interfere in the Ca^2+^-switch of LPL as we expected (Fig. 4e).

In addition to the Ca^2+^ switch, the targeting of LPL to the actin cytoskeleton is also regulated by the phosphorylation of Ser5^41^. Among all three human plastin isoforms, LPL is the only isoform that is currently known to be phosporylated *in vivo*^40^. Unlike other actin-binding proteins, where phosphorylation usually occurs in the actin-binding domain, LPL phosphorylation seems to take place on the regulatory Ca^2+^-binding domain. Janji and coworkers demonstrated that compared to the non-phosphorylated LPL protein, the S5E mutant had increased actin bundling activity^41^. They hypothesized that Ser5 phosphorylation would cause a conformational change in the Ca^2+^-binding domain that would affect the interaction of the actin-binding domains with actin. Our results show that the phosphorylation of Ser5 does not induce a conformational change in the EF domain. From the reduced actin-bundling observed with H5-ABD12 construct compared to the full-length LPL, it is clear that the EF domain also contributes to the actin bundling (Fig. 4a,b), consistent with a previous report^61^. Therefore, we propose that the phosphorylation of Ser5 directly enhances this effect.

Development of metastasis provides the most serious challenge for cancer treatment and is responsible for most cancer related deaths. A number of recent studies have shown that ectopically expressed LPL is largely responsible for this process^19^. Therefore, LPL presents itself as a promising target for drug development to prevent the metastatic activity of the cancer cells. It is also well known that inhibition of fascin, another actin-bundling protein, is a viable approach to block tumor metastasis^66^,^67^. In this study, as a proof of concept, we have demonstrated that melittin that can interfere with the association of the EF-domain with H5 can disable the Ca^2+^-switch of LPL. The melittin peptide, which is isolated from bee venom, has been reported to have anticancer activity including the inhibition of metastasis by reducing cell motility and it has been proposed as an agent for anticancer therapy^68^,^69^. However, melittin is also a strong hemolytic peptide that is toxic to all normal cells. Therefore, it is not a suitable peptide for direct clinical applications. Nevertheless, drugs that can block the binding pocket of the EF domain of LPL and that are safe for clinical application could be discovered via high-throughput screening. Therefore, we believe that our findings may be a significant first step towards the future development of drugs that target the metastatic events that occur in many types of cancer cells.

## Methods

### Protein expression and purification

A synthetic gene with optimized codons for the expression of the full-length human L-plastin protein (LPL) in *E. coli* was purchased from GeneArt. From the full-length LPL gene as the template, all the constructs used in this study were generated by a standard PCR protocol (Fig. 1). To generate the expression vectors for the LPL-EF, LPL-EF-H5, ABD1, H5-ABD1, and ABD2 constructs, the PCR products were subcloned into a pET15 vector (Invitrogen), which contained an N-terminal His-tag and a TEV protease cleavage site, using *NdeI* and *XhoI* sites. To generate the expression vectors for all other constructs, the PCR products were subcloned into a pGEX-6p-1 vector (GE Healthcare) using *XhoI* and *BamHI* sites. The vector was modified so as to contain a TEV protease cleavage site between the GST and construct. To create EF-H5-S5E, which mimics the phosphorylated state, Ser 5 near the N-terminal end of the EF-H5 construct was mutated to Glu using the Quick Change site-directed mutagenesis kit (Stratagene). All the recombinant plasmids were transformed into competent *E. coli* BL21 (DE3) (Novagen) cells for protein expression.

*E. coli* cells were grown in Luria Bertani medium with 100 μg/ml of ampicillin at 37 °C. Uniformly ^15^N- or ^15^N,^13^C-labelled proteins were prepared in M9 minimal medium supplemented with 0.5 g/L ^15^NH_4_Cl and/or 3 g/L ^13^C-glucose. At an optical density of ~0.6 (600 nm), the cultures were induced with 0.5–1.0 mM IPTG. After 4 hours, the bacterial cells were harvested by centrifugation. For the His-tagged constructs, the cell pellet was resuspended in the IMAC binding buffer (20 mM Tris-Cl, 0.1 M NaCl, and 50 mM imidazole, pH 8.0) and lysed via French Press. The supernatant was applied onto an IMAC column (GE Healthcare). The column was washed extensively with the IMAC binding buffer and the His-tagged proteins were eluted with the elution buffer (20 mM Tris-Cl, 0.1 M NaCl, and 300 mM imidazole, pH 8.0). The His-tag was then cleaved with TEV protease in the digestion buffer (20 mM Tris-Cl, 0.1 M NaCl, 0.5 mM EDTA, and 1 mM dithiothreitol) at 34 °C. The mixture was then loaded onto the cOmplete column (GE Healthcare) to remove the TEV protease and the His-tag from the protein. The GST-fusion proteins were purified using a Glutathione Sepharose 4 Fast Flow column (GE Healthcare). The cell pellet was resuspended in the extraction buffer (50 mM Tris-Cl, 100 mM NaCl, 1 mM EDTA, pH 7.5) and lysed by French Press. The supernatant was applied onto the column equilibrated with the extraction buffer. The column was washed with the extraction buffer supplemented with 0.5% Triton X-100 and then further washed with the elution buffer with 0.1% Triton X-100. The column was equilibrated with the digestion buffer and the GST-fusion protein was digested with a TEV protease on the column at 34 °C. The eluted fractions were passed through the cOmplete column to remove the TEV protease. TEV protease was expressed and purified from the pRK793 plasmid as previously described^70^. Protein purification was verified using SDS-PAGE and Coomassie brilliant blue staining.

### NMR spectroscopy experiments

The calcium-free samples of EF-H5, EF-H5-S5E, and EF contained 0.5–1.0 mM ^15^N- or ^13^C,^15^N-labeled protein, 100 mM KCl, 20 mM Bis-Tris (pH 6.8), 1 mM EDTA, 0.03% NaN_3_, 10 mM ^2^H-labelled dithiothreitol (^2^H-DTT), and 0.5 mM 2,2-dimethyl-2- silapentane-5-sulfonate (DSS) in 99.99% D_2_O or 10% D_2_O/90% H_2_O. The calcium free non-labeled LPL-EF was also prepared in 99.99% D_2_O with the same buffer components. The calcium-bound form of those constructs were prepared in buffer containing ~1 mM ^15^N- or ^13^C,^15^N-labelled protein, 100 mM KCl, 20 mM Bis-Tris (pH 6.8), 5 mM CaCl_2_, 0.03% NaN_3_, 0.5 mM DSS, and 10 mM ^2^H-DTT in 99.99% D_2_O or 10% D_2_O/90% H_2_O. The calcium-bound non-labeled LPL-EF-H5 was also prepared in 99.99% D_2_O with the same buffer ingredients. DSS was used as a reference to obtain ^1^H, ^15^N and ^13^C chemical shifts. All NMR spectra were processed with NMRPipe^71^ and analyzed using NMRView^72^.

All NMR experiments were performed with a Bruker Avance 700 or 600 MHz spectrometer at 25 °C. Main-chain NMR signal assignments of the calcium-bound and the calcium-free LPL-EF-H5, and calcium-free LPL-EF were completed with two-dimensional ^1^H,^15^N-HSQC and three-dimensional HNCACB, CBCA(CO)NH, HN(CA)CO, HNCO, HNCA, and HN(CO)CA experiments. Aliphatic side-chain assignments were obtained by three-dimensional C(CCO)NH-TOCSY, H(CCO)HN-TOCSY, and HBHA(CBCACO)NH experiments. Aromatic side-chain assignments were achieved using two-dimensional (HB)CB(CGCD)HD and (HB)CB(CGCDCE)HE experiments. All NOESY experiments including three-dimensional ^15^N-edited NOESY-HSQC and ^13^C-edited NOESY-HSQC, and two-dimensional NOESY were measured with a mixing time of 120 ms. {^1^H}-^15^N heteronuclear NOE data were obtained using a 5 sec train of 120° proton pulses.

### Structure calculations

CYANA version 2.0^73^ was used to calculate the structure of the calcium-free EF and the calcium-bound EF-H5. This was done using distance restraints generated from the automated NOE assignment protocol implemented in CYANA. The dihedral angle restraints were predicted by TALOS+^74^, and hydrogen bond restraints for α-helices were based on the secondary structure derived from the chemical shift index for the Cα and C’ atoms. A total of 12 Ca^2+^-ligand restraints for the calcium-binding loops were also introduced according to the well-known Ca^2+^ coordination geometry^53^,^54^. The 30 lowest-energy structures from a total of 200 were used for analysis. The program MOLMOL was used to generate the molecular graphics^75^.

### Isothermal titration calorimetry (ITC)

All ITC experiments were carried out on a Microcal VP-ITC microcalorimeter. 40 µM LPL-EF or LPL-EF-H5 in calcium-free buffer containing 20 mM HEPES (pH 7.2), 100 mM KCl, and 1 mM Tris(2-carboxyethyl)phosphine, was injected with 1.2 mM CaCl_2_ in the same buffer at 25 °C. Prior to each titration, the ITC sample cell was soaked in 5 mM EDTA solution, and was rinsed stringently afterwards with calcium free buffer. The calcium free buffer was prepared via 1 week incubation with Chelex chelating agent. Each protein sample was exchanged into the calcium free buffer, and then passed through a Calcium Sponge S column (Life Technologies). Data was fitted using the one or two site model, as applicable, with the MicroCal Origin software to obtain the stoichiometry (N), affinity (K), free enthalpy (ΔH), and entropy (ΔS) values.

### Surface plasmon resonance (SPR)

The binding between Ca^2+^-bound LPL-EF and a synthetic peptide corresponding to H5 (Ac-STDVAKTFRKAINKKEGI-NH2) was evaluated by SPR using a BIAcore X100 instrument (GE Healthcare) and was compared to other peptides including the cytotoxic peptide melittin, and the CaM-target CaMKI and smMLCK peptides. Peptides used here were purchased as synthetic peptides with >95% purity from Genscript (San Diego, CA). The EF construct was immobilized via its sole cysteine residue (Cys42) onto a CM5 sensor chip (GE Healthcare) using thiol-coupling. The running buffer contained 10 mM Tris-HCl pH 7.5, 150 mM KCl, 1 mM CaCl_2_, and 0.005% (v/v) Tween-20. Different concentrations of the peptide sample were injected at a flow rate of 30 μL/min with a contact time of 1 min at 25 °C. The BIAevaluation software 2.0 (GE Healthcare) was used to process the SPR sensorgrams and for curve-fitting to obtain the dissociation constants (*K*_*d*_’s). Two different concentrations in each experiment were injected twice to obtain the fitting errors (SEM).

### Actin-bundling assay

Human platelet actin (Cytoskeleton) was dissolved in 2 mM Tris pH7.5, 0.2 mM CaCl_2_, 0.2 mM ATP, and 0.5 mM DTT (Buffer G). The actin was then polymerized overnight at 4 °C in 20 mM Tris pH 7.5, 100 mM KCl, and 1 mM MgCl_2_. Spontaneously bundled F-actin was removed by centrifugation at 12,000 g for 15 min prior to the assay. F-actin and LPL constructs were incubated at room temperature for 1 h in 50 uL of the buffer containing 20 mM Tris pH7.5, 100 mM KCl, 1 mM MgCl_2_, 6 µM F-actin, 3 µM LPL construct, 0.1 mM CaCl_2_, 0.1 mM ATP with and without 5 mM EGTA. LPL induced actin bundles were sedimented by centrifugation at 12,000 g for 15 min and the supernatant was carefully removed. The amount of actin and/or LPL construct in the pellet was analyzed by SDS-PAGE. The effect of the presence of 12 µM melittin peptide on actin-bundling was also examined.

### Data Availability

The atomic coordinates, NMR constraints and resonance assignments have been deposited in the Protein Data Bank (PDB IDs 5JOJ and 5JOL) and the BMRB data base (BMRB-30071 and 30072).

## Additional Information

**How to cite this article**: Ishida, H. *et al*. The Calcium-Dependent Switch Helix of L-Plastin Regulates Actin Bundling. *Sci. Rep.* **7**, 40662; doi: 10.1038/srep40662 (2017).

**Publisher's note:** Springer Nature remains neutral with regard to jurisdictional claims in published maps and institutional affiliations.

## Supplementary Material

Supplementary Information

## Figures and Tables

**Table 1:** ITC derived thermodynamic parameters for the Ca^2+^-binding to EF-H5 and EF.

Constructs	N1	K_d_1 (µM)	H1 (cal/mol)	S1 (cal/mol/K)	N2	K_d_2 (µM)	H2 (cal/mol)	S2 (cal/mol/K)
EF-H5^1^	2.3	0.7 ± 0.2^2^	−4507 ± 56	13.0 ± 0.2	—	—	—	—
EF	1.0	0.3 ± 0.1	−1341 ± 82	24.3 ± 2.0	1.2	2.6 ± 0.2	−4179 ± 236	10.8 ± 1.9

^1^Analyzed as a single binding event. The isotherm contained two binding events that are too similar to be differentiated unambiguously.

^2^Average values and SD values were obtained from three independent ITC runs (*n* = 3).

**Table 2:** Determination of the dissociation constants between Ca^2+^-bound EF and various peptides by surface plasmon resonance.

Peptide	*K*_*D*_ (M)
H5p	(3.52 ± 0.24) ×10^−6^
CaMKIp	(3.27 ± 0.15) ×10^−6^
Melittin	(1.58 ± 0.20) ×10^−7^
