# Supplementary material for: The Calcium-Dependent Switch Helix of L-Plastin Regulates Actin Bundling
Source: Sci Rep. 2017 Feb 1;7:40662. doi: 10.1038/srep40662 (PMC5286426; doi:10.1038/srep40662)
Supplement: Supplementary Information [file srep40662-s1.pdf]

The Calcium-Dependent Switch Helix of L-Plastin Regulates Actin Bundling

Hiroaki Ishida, Katharine V. Jensen, Andrew G. Woodman, M. Eric Hyndman and Hans J. Vogel

Supplementary Table S1 | Experimental restraints and structural statistics of the 30 NMR structures.

	EF	EF-H5
**Number of experimental restraints**		
Distance restraints from NOE		
Total	2166	2662
Sequential	1400	1663
Medium range (2-4)	311	484
Long range (5+)	455	515
Dihedral angle restraints (TALOS)	138	158
Hydrogen bond distance restraints	60	72
Calcium-ligand distance restraints	0	12
**PROCHECK Ramachandran analysis of folded regions (%)**		
Residues in favored regions	89.0	90.5
Residues in additional allowed regions	10.2	9.5
Residues in generously allowed regions	0.8	0.0
Residues in disallowed regions	0.0	0.0
**Coordinate precision of folded regions (Å)**		
Backbone	0.40 ± 0.12	0.37 ± 0.08
All heavy atoms	0.90 ± 0.10	0.91 ± 0.09

Supplementary Figure S1

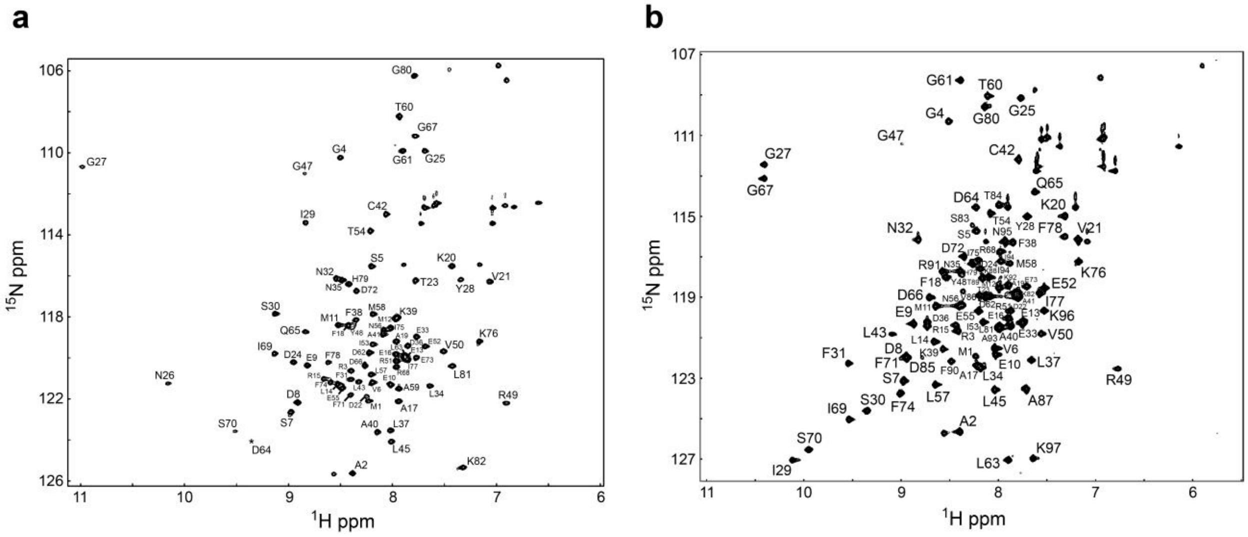

The assigned $^{1}H,^{15}N$ HSQC NMR spectra of (**a**) Ca^{2+}-free EF and (**b**) Ca^{2+}-bound EF-H5. Both spectra were acquired at 25 °C.

Supplementary Figure S2

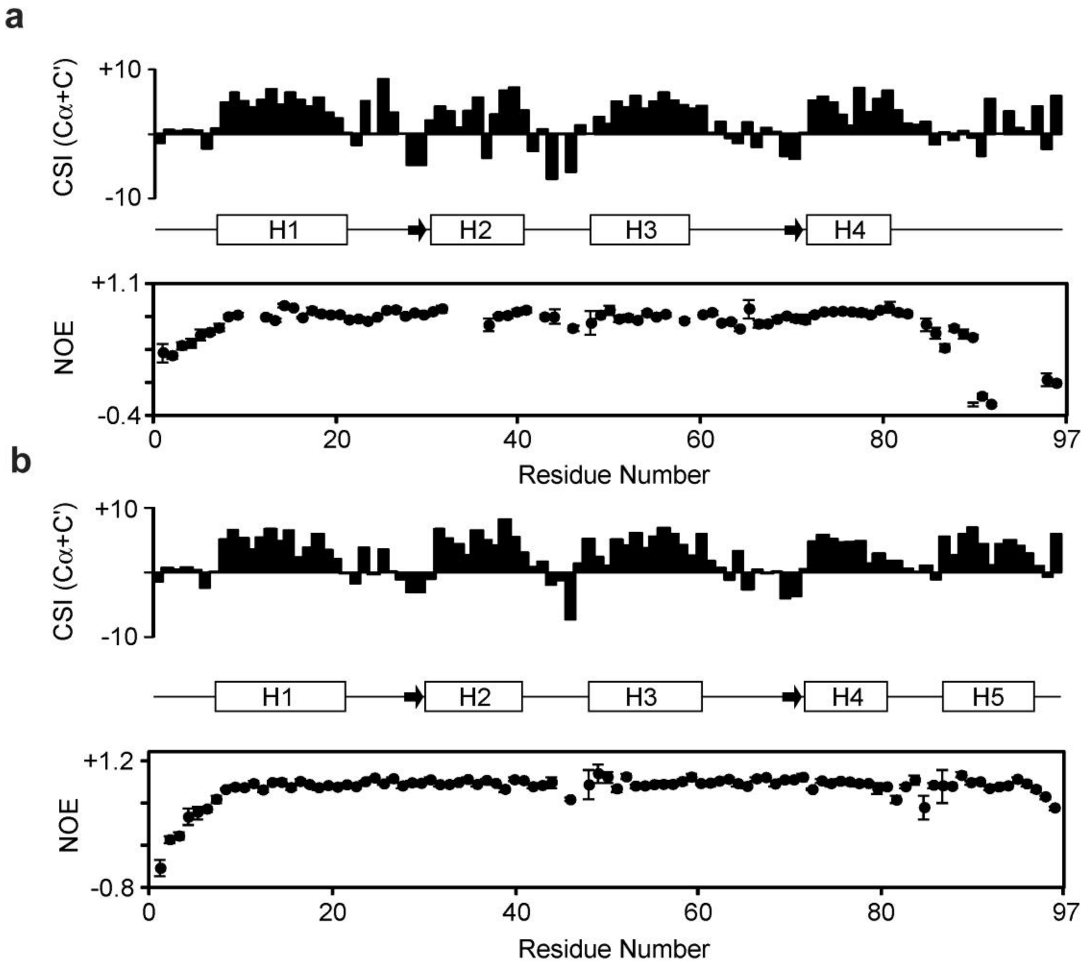

Structural NMR data obtained for EF-H5 in the (**a**) Ca^{2+}-free and (**b**) Ca^{2+}-bound forms. CSI values for C_α and C' atoms (top panel). The backbone {^{1}H}, ^{15}N-NOE (bottom panel). The positions of the secondary structure elements are also shown. The boxes and arrows indicate helices and sheets, respectively.

Supplementary Figure S3

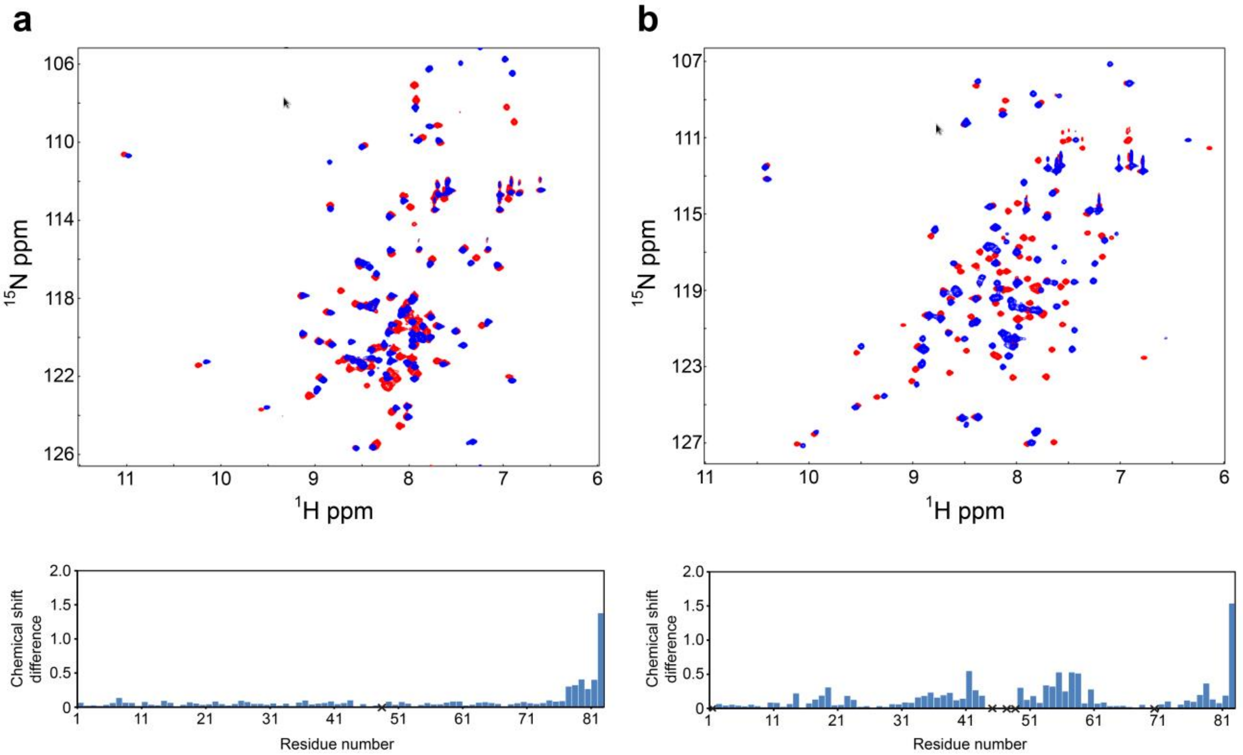

Overlaid $^{1}H,^{15}N$ HSQC NMR spectra of EF (blue) and EF-H5 (red) (top) and the normalized chemical shift differences plotted as a function of residue number (bottom) for the Ca^{2+}-free (**a**) and the Ca^{2+}-bound (**b**) forms. In the bottom panels, the residues marked with a cross indicate the data not available. In the case of the apo-form, judging from the chemical shift perturbations, there are clearly no substantial interactions between the H5 region and the two EF-hand motifs of LPL, while the opposite is true for the Ca^{2+}-form.

Supplementary Figure S4

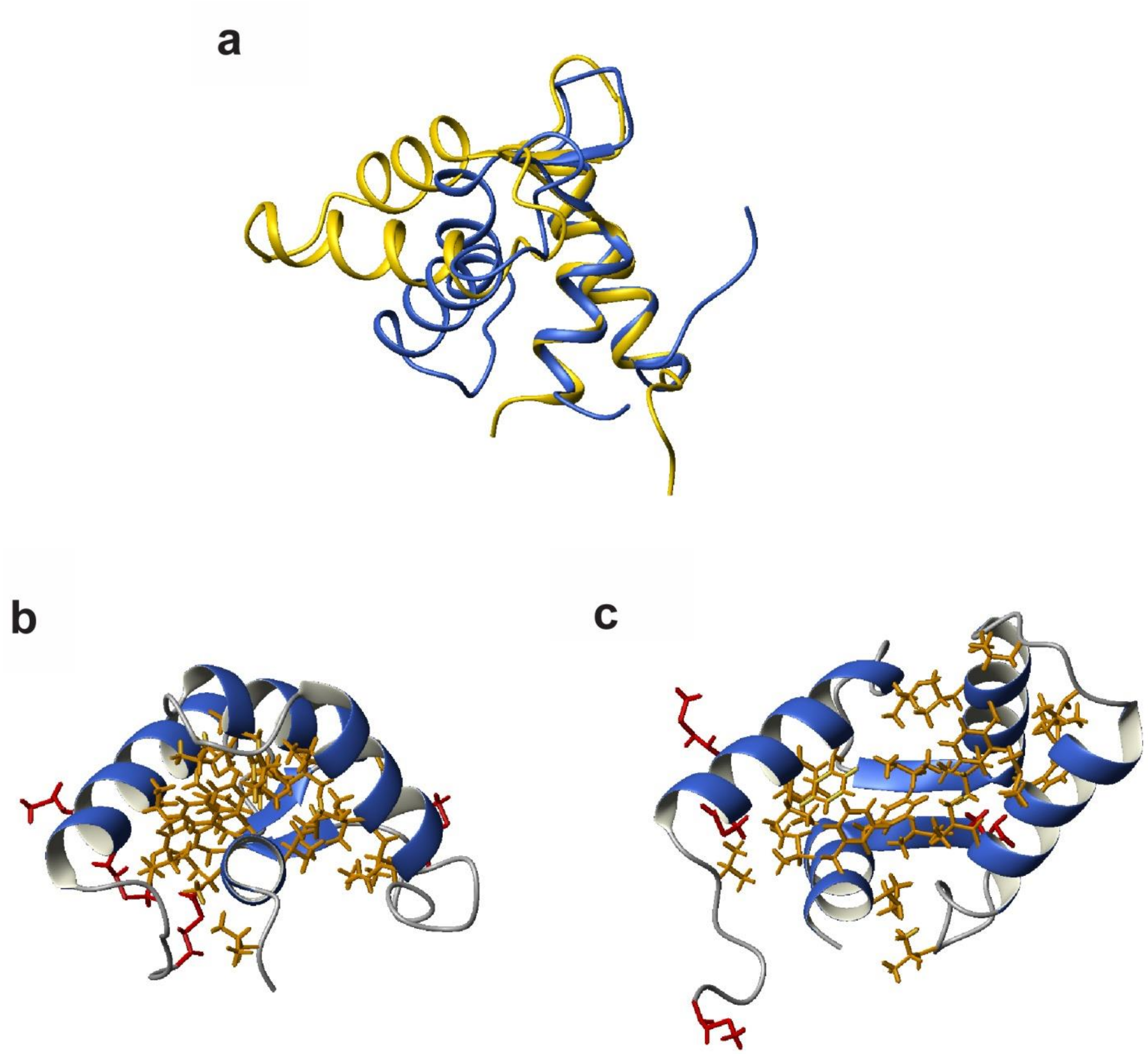

(**a**) Overlaid structures of Ca^{2+}-free and Ca^{2+}-bound EF-hands of LPL. Structures are overlaid using helix 1 and 4 to emphasize the reorientation of helix 2 and 3. The hydrophobic core of the EF-hand domain of LPL in the Ca^{2+}-free (**b**) and Ca^{2+}-bound (**c**) forms. Methionine side-chains are shown in red, whereas all other hydrophobic residues are shown in orange.

Supplementary Figure S5

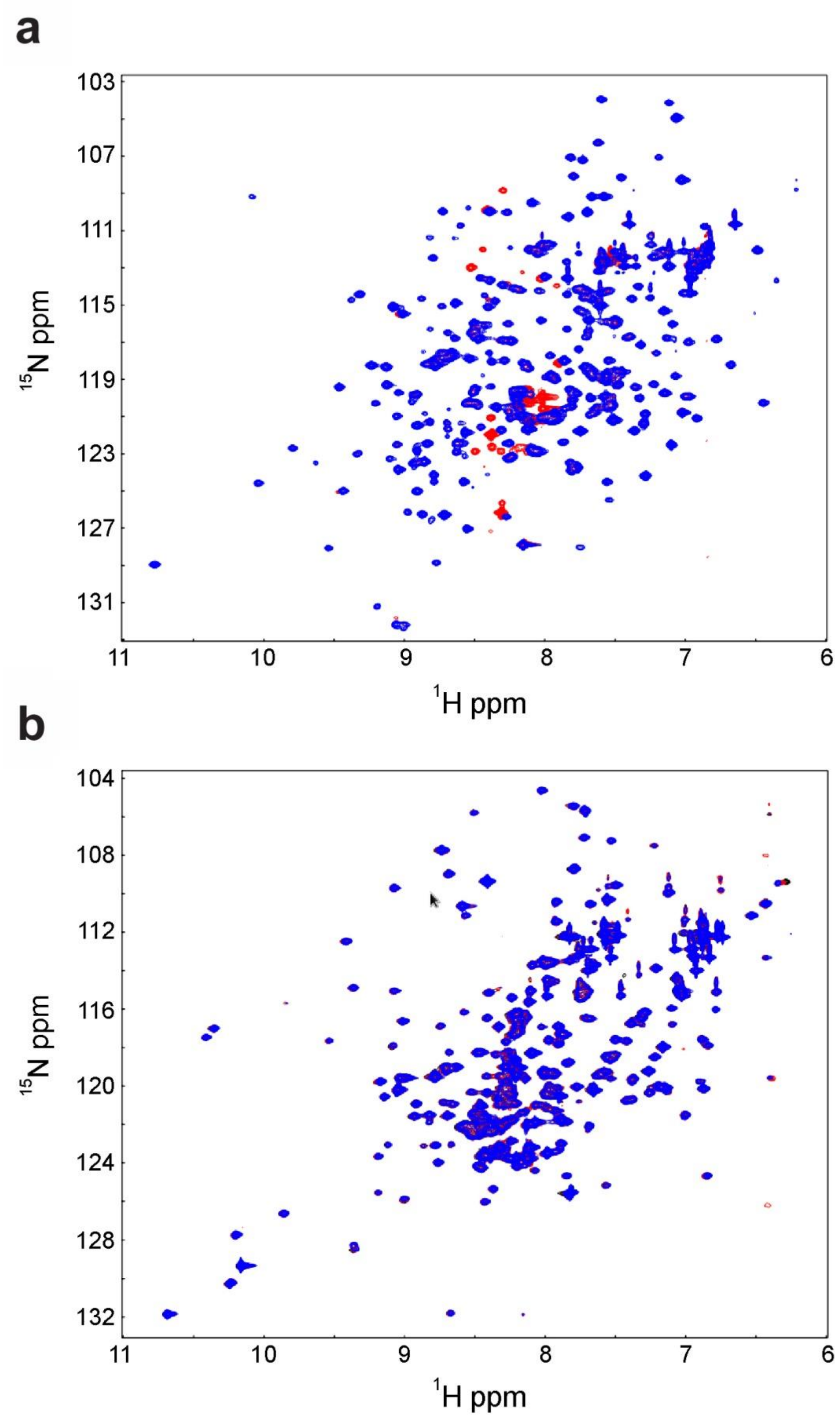

(**a**) Overlaid ^{1}H,^{15}N-HSQC NMR spectra of ABD1 (blue) and H5-ABD1 (red). (**b**) The synthetic H5 peptide was titrated into ABD2. The spectra with 0, 1, and 2 molar excess of H5 peptide are shown in black, red, and blue, respectively.

Supplementary Figure S6

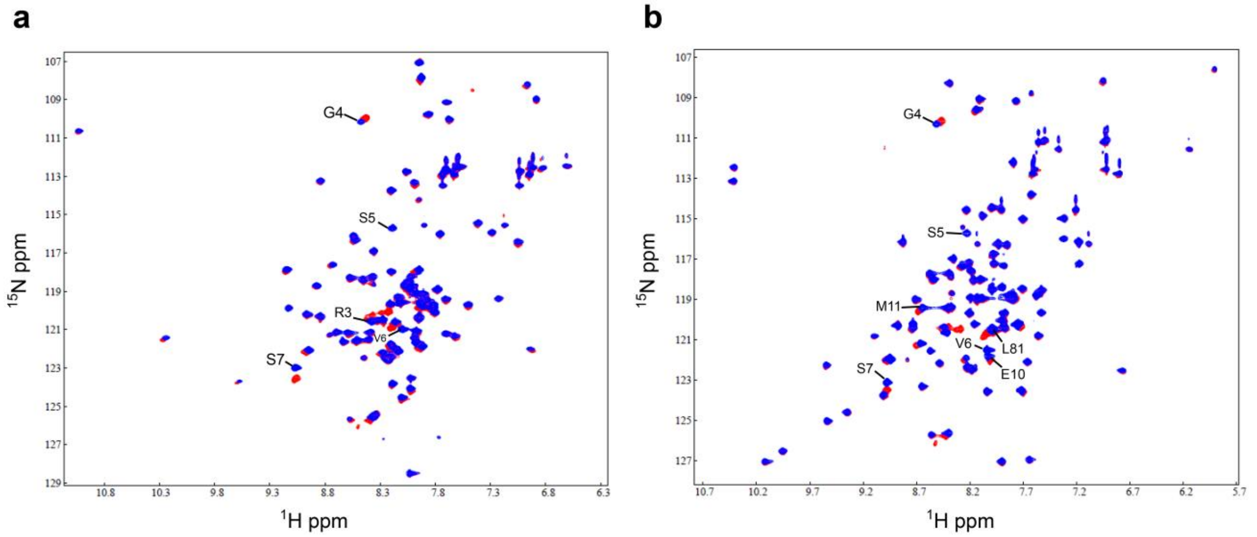

The ^{1}H,^{15}N-HSQC NMR spectra of EF-H5 (blue) and EF-H5-S5E mutant (red) in the presence (**a**) and absence (**b**) of Ca^{2+} are overlaid.

Supplementary Figure S7

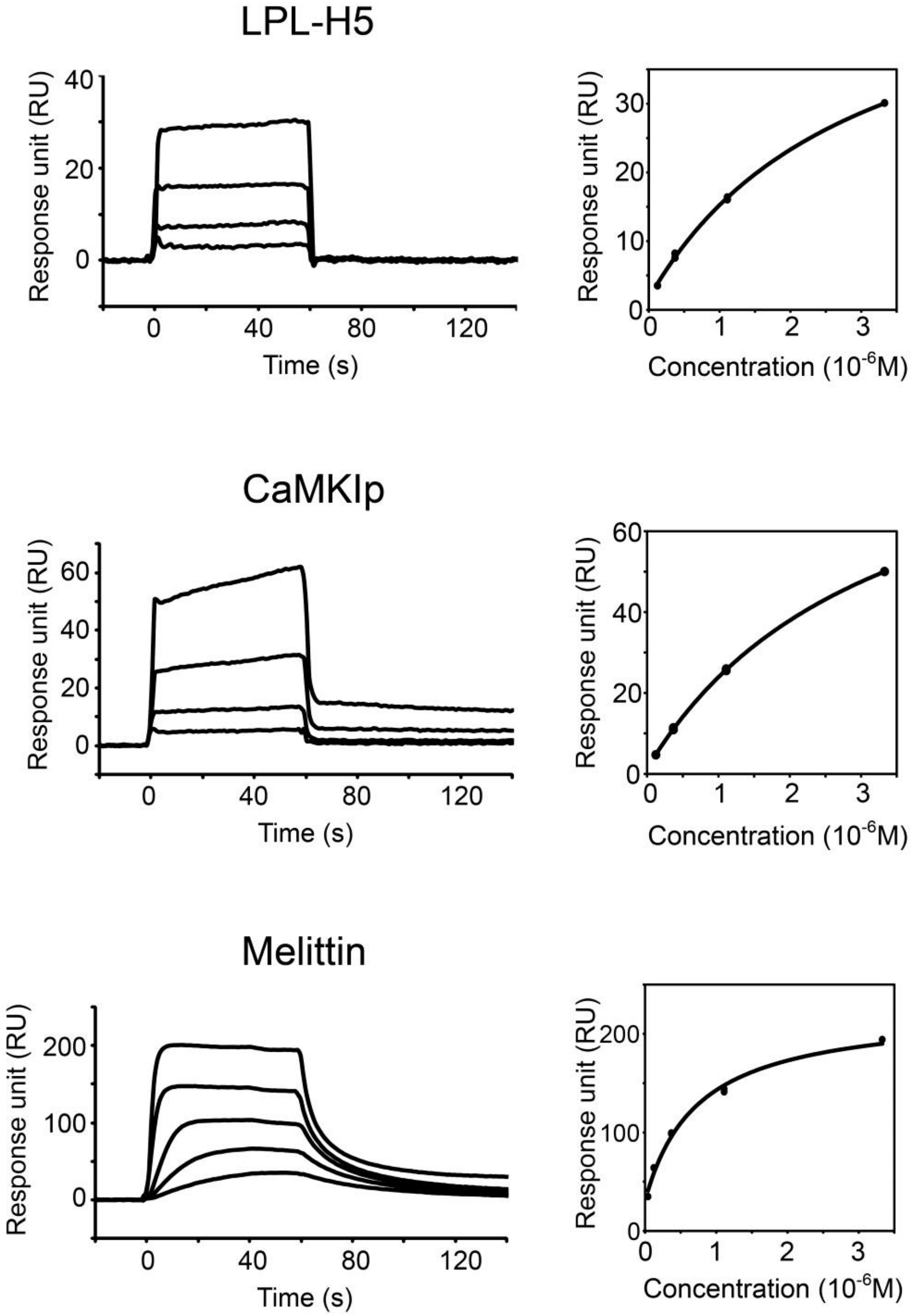

SPR measurements for the interactions between EF and various peptides. The affinity fittings used for the determination of the Kd are also displayed. The response units at 4 seconds after starting the injection were used for CaMKIp due to the nonspecific binding seen later during the injection, while the response units at 4 seconds before stopping the injection were used for the other peptides.

Supplementary Figure S8

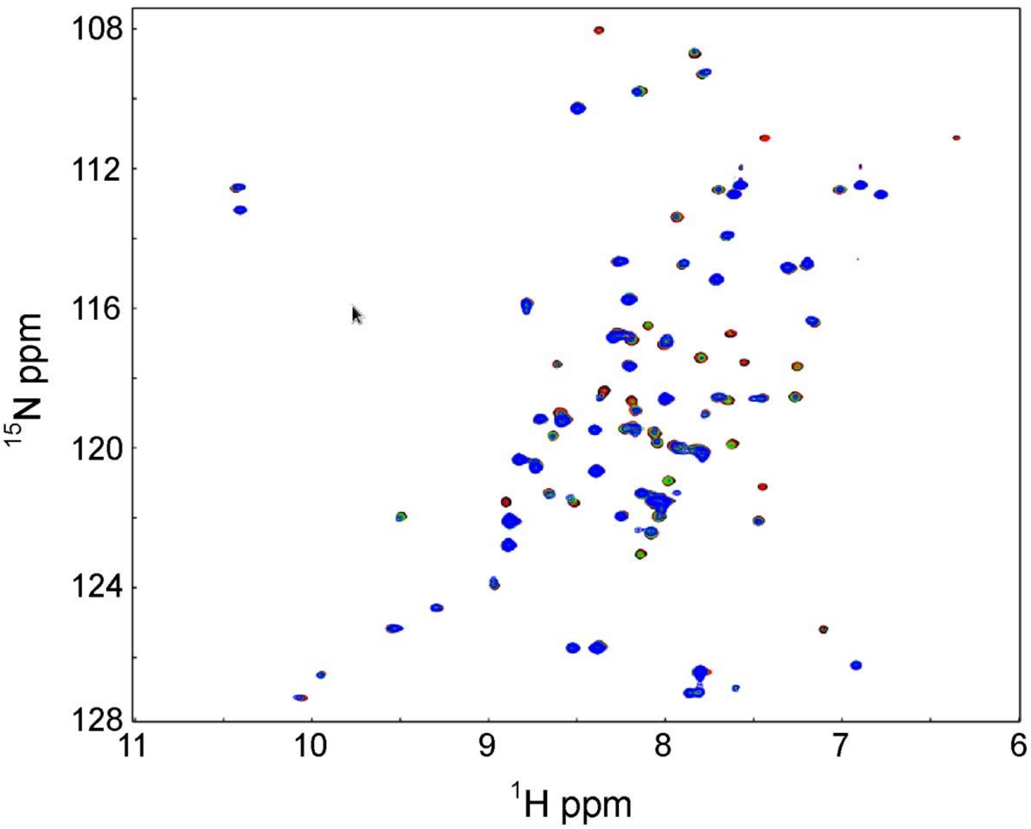

$^{1}H,^{15}N$-labeled EF was titrated with the H5 peptide. The $^{1}H,^{15}N$-HSQC NMR spectra with 0, 0.5, 1.0, and 1.5 equivalents added are shown in black, red, green, and blue, respectively. The spectral changes represent intermediate exchange on the NMR time scale.

Supplementary Figure S9

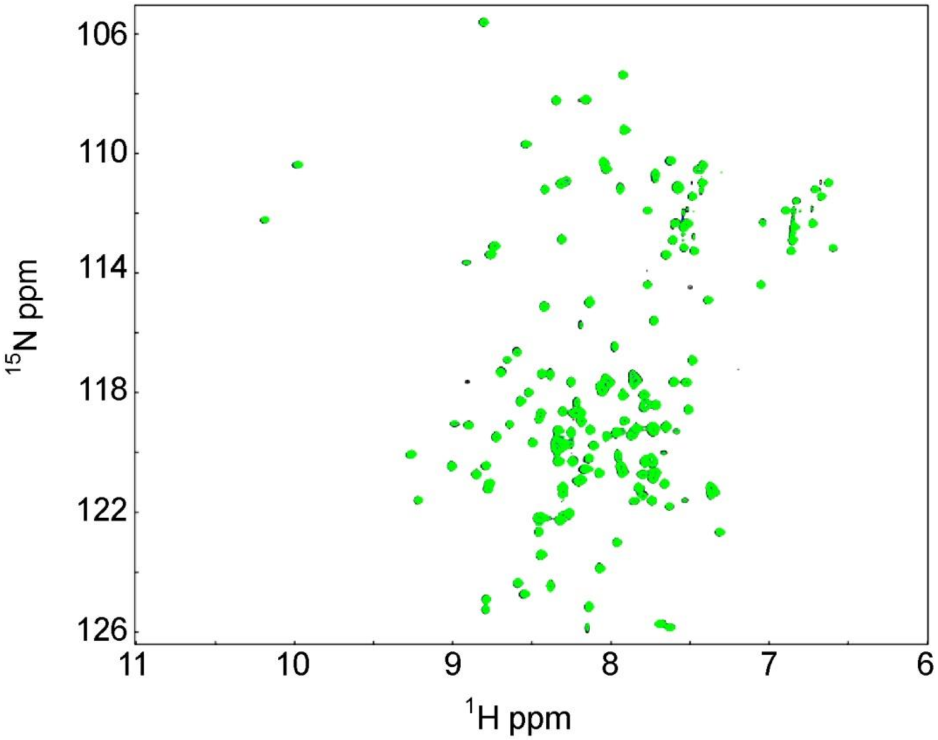

Overlaid ^{1}H,^{15}N-HSQC NMR spectrum of Ca^{2+}-free CaM with (green) and without (black) 1.2 molar excess of unlabeled EF-H5 in the absence Ca^{2+}.
